# The Use of *Euterpe oleracea* Mart. As a New Perspective for Disease Treatment and Prevention

**DOI:** 10.3390/biom10060813

**Published:** 2020-05-26

**Authors:** Thalita Sévia Soares de Almeida Magalhães, Pollyana Cristina de Oliveira Macedo, Attilio Converti, Ádley Antonini Neves de Lima

**Affiliations:** 1 Department of Pharmacy, Laboratório Escola de Farmácia Industrial, Federal University of Rio Grande do Norte, Natal RN 59012-570, Brazil; thalitasevia22@gmail.com (T.S.S.d.A.M.); macedopollyanax@gmail.com (P.C.d.O.M.); 2 Department of Civil, Chemical and Environmental Engineering, Pole of Chemical Engineering, Genoa University, I-16145 Genoa, Italy; converti@unige.it

**Keywords:** *Euterpe oleracea* Mart., açaí, pharmacological activity, systematic review, patents

## Abstract

*Euterpe oleracea* Mart. (EO), popularly known as açaí, belongs to the Arecaceae family and grows abundantly in Brazil. The fruit of this palm tree is widely used because of its anti-inflammatory and antioxidant properties. In this review, a search for literature and patent technological prospecting has been performed on the use of EO to treat and prevent diseases as well as to prepare pharmaceutical formulations. EO leaves, fruits, and oil stand out for their large number of pharmacological activities such as anti-inflammatory, antioxidant, antimicrobial, antinociceptive, anticancer, anti-atherogenic, and healing activities, protection against metabolic syndromes such as diabetes, hypertension, and hyperlipidemia, and protection of organs such as lung, kidney, liver, heart, and nervous system. While the phytochemical composition is intrinsically linked to identified biological activities, discoveries of the past decade concerning the use of this species have shown pharmacological alternatives mainly in the treatment and prevention of breast cancer and metabolic syndromes. Although studies and inventions on the use of EO though are believed to have been important in light of the pharmacological activities found, few clinical and toxicity tests have been performed. Nevertheless, with the increase of interest in EO, this species is believed to be only at the beginning of the breakthroughs in the development of promising products for the pharmaceutical industry.

## 1. Introduction

The use of plants for therapeutic and healing purposes has been a habit all over the world since primordial times [1,2]. With the rise of allopathic medicines, in the 1930s and 1940s there was a decrease in incentives for the use of plant extracts, reducing the initiative for studies to demonstrate their efficacy and safety [3].

However, most pharmaceutical products are known to consist of metabolites isolated from plants [4], which drive “alternative medicine”. Bruning et al. [3] also reported that the interest in the search for new pharmacological alternatives among plants dates back to the 1970s, 1980s, and especially the 1990s, thus giving rise to phytotherapy [5]. This activity is maintained to the present day, constituting the traditional medicine of many cultures [6,7,8].

The growth in sales of herbal supplements and pharmaceutical formulations occurs mainly in Europe and Asia-Pacific, while Brazil remains the country with the greatest plant diversity [9,10]. Brazilian rainforest does in fact hold great biodiversity, containing approximately 23% of the existing plant species worldwide [11]. This biome is relevant to the pharmaceutical industry, since physicochemical and pharmacological studies enable the discovery of important biological activities, suggesting the development of new products from native species.

Plants of the genus *Euterpe* have deeply been studied. Santamarina et al. reported the anti-inflammatory and hepatoprotective effects of the pulp of *Euterpe edulis* Mart. [12,13], while Cižauskait et al. [14] evaluated the use of açaí as a dye and an enhancer of product sensory properties. The species *Euterpe oleracea* Mart. (EO), popularly known as “açaí”, is widely consumed because of its antidiarrheal, anti-inflammatory and antioxidant properties [15,16,17,18], but numerous studies have also shown antinociceptive [19], anticonvulsant [20], antioxidant [21], antiangiogenic [22], antimicrobial [23,24,25], antileishmania [26], anti-aging [27], and neuroprotective [28] activities related to extracts and fruit oil. In this context, the present study aims to develop a literature review and technological prospecting for patents on the use of EO as a pharmacological alternative for the treatment and prevention of diseases.

## 2. *Euterpe oleracea* Mart. Plant Phytochemical Composition

**Figure 1:**
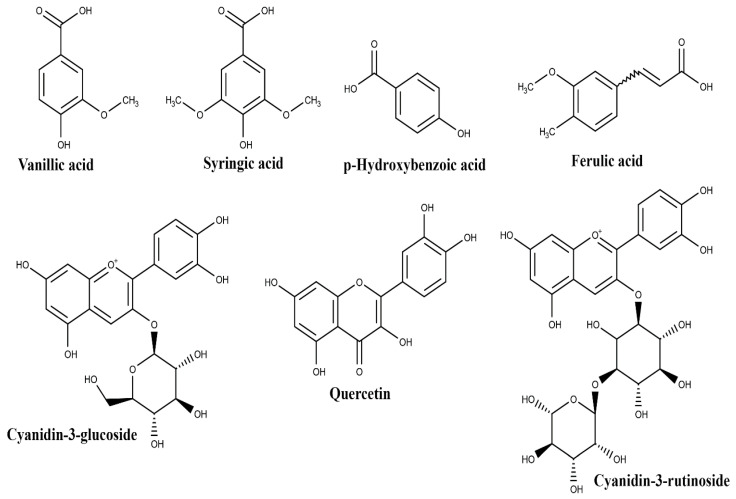
Structures of the main compounds identified in *Euterpe oleracea* Mart. fruit.

Interest in EO is currently increasing not only because of its wide use in the food and cosmetic industries, but also because of its potential in the pharmaceutical sector [17]. Different parts of EO such as fruits, leaves, roots, and fruit oil have in fact been studied for pharmacological application, suggesting different biological activities according to their chemical composition. A variety of phenolic acids, among which are vanillic, syringic, *p*-hydroxybenzoic, protocatechuic, and ferulic acids, as well as (+)-catechin and procyanidin oligomers, have been identified in high concentrations in EO pulp and oil extracts [29] (Figure 1 and Table 1).

Pacheco-Palencia et al. [29] states that extracts enriched with these compounds are responsible for antioxidant activity. Moreover, Agawa et al. [30] and Poulose et al. [31] reported the presence of the flavonoid quercetin and anthocyanins, such as cyanidin-3-glucoside and cyanidin-3-rutinoside (Figure 1 and Table 1) in EO extracts, confirming their antioxidant activity. Poulose et al. [31] also reported an anti-inflammatory effect, corroborating the results of Kang et al. [32], Cedrim et al. [33], and Mathias et al. [34].

Several studies performed on EO fruit extracts and oil have shown other biological activities, among which are the antinociceptive [19], antiangiogenic [22], antimicrobial [23,24,25], antileishmanial [26], anticonvulsant [35], and antiproliferative [36] ones, which suggested their application in the treatment and prevention of metabolic syndromes such as cardiovascular disease, diabetes and dyslipidemias [37,38,39,40]. They are listed in Table 2 and discussed in Section 3.1, among other diverse biological activities.

**Figure 2:**
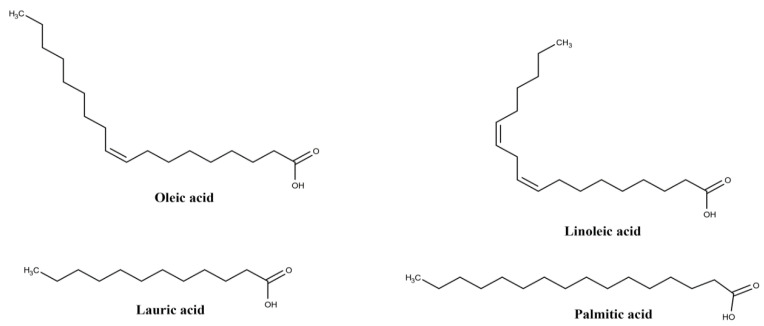
Structures of the main fatty acids in *Euterpe oleracea* Mart. oil.

EO oil, which is extracted from the pulp or seed of the fruit, consists mainly of fatty acids, whose major constituent is oleic acid (47.58%), followed by palmitic (24.06%) and linoleic (13.58%) acids (Figure 2), while palmitoleic, vaccenic, lauric and stearic acids were detected in minor concentrations [25]. Melhorança Filho and Pereira [23] and Magalhães et al. [25] ascribed significant antibacterial activity, Favacho et al. [19] anti-inflammatory and antinociceptive effects, and Souza et al. [40] antilipemic action to the oil. A study by Pacheco-Palencia et al. [29] also reported the presence of polyphenolic compounds and anthocyanins retained in the oil, among which are procyanidin dimers, procyanidin trimers, vanillic acid, and syringic acid (Table 1), which contribute to its significant antioxidant activity.

**Figure 3:**
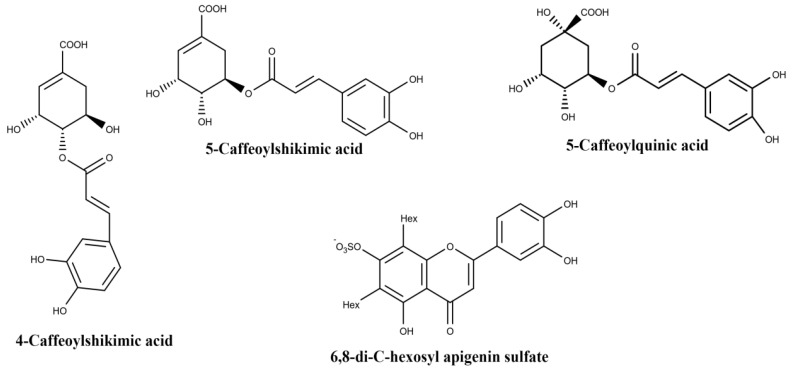
Structures of the main components of *Euterpe oleracea* Mart. root and leaf; Hex: hexose.

A phytochemical study on EO root and leaf extracts pointed out high concentrations of hydroxycinnamic acids such as caffeoylquinic acids, caffeoylshikimic acids, and flavone derivatives such as C-glycosyl derivatives of apigenin and luteolin. Table 1 lists the main compounds identified in the respective extracts. Therein, 5-Caffeoylquinic acid, 4-caffeoylshikimic acid and 5-caffeoylshikimic acid stand out in root extracts, while 5-caffeoylquinic acid and 6,8-di-C-hexosyl apigenin sulfate, which has a sulfate in position 7 or on the sugar moieties, stand out in the leaf extract. Figure 3 shows their molecular structures [41]. These authors highlighted the interest of the pharmaceutical, cosmetic, and food industries, especially for leaves, due to their antioxidant potential.

## 3. Biological EO Activities: Application in the Prevention and Treatment of Diseases

EO has been long used in folk medicine. Plotkin and Balick [15] reported its empirical use against diarrhea. Only in 2002 did a study on EO leaf and steam extracts report a reduction in abdominal contortions and peripheral analgesic action [42], while in 2004 and 2005 the antioxidant capacity of EO fruit was attributed to flavonic compounds present in its extract [43,44]. Over the years, several studies have reported pharmacological applications related to biological activities of this species. The biological activities found in the literature are summarized in Table 2 together with the respective pharmacological applications.

### 3.1. Pharmacological Applications of Euterpe oleracea Mart. Fruit

#### 3.1.1. Pro-Apoptotic Effect

A polyphenolic fraction obtained from juice was tested against human colorectal adenocarcinoma (HT-29), colon adenocarcinoma (SW-80) and colon fibroblast cells (CCD-18Co). The extract inhibited the growth of SW-480 cells more than that of HT-29 cells but had no toxicity to the non-malignant CCD-18Co cells. The mechanisms involved in colon cancer cell growth suppression include protection against reactive oxygen species (ROS) production and downregulations of nuclear factor-kappa B (NF-kB), NF-kB-target vascular cell adhesion molecule-1 (VCAM-1), intercellular adhesion molecule-1 (ICAM-1), Sp prooncogenic transcription factors, Sp-target vascular endothelial growth factor (VEGF), Bcl-2, and survivin. It also activated the mitochondrial pro-apoptotic pathway that resulted in cytochrome c release, caspase-3 cleavage, and poly (ADP-ribose) polymerase-1 (PARP-1) degradation [45].

#### 3.1.2. Anticancer Effect

In vitro and in vivo studies using different parts of EO have pointed out anticancer effects against different cell lines (Table 2). Choi et al. [46] observed that the administration of pellets containing 5% of lyophilized EO berries reduced significantly the incidence of both adenoma (from 76.9% to 23.1%) and cancer (from 76.9% to 15.4%) as well as the expressions of myeloperoxidase (MPO) and proinflammatory cytokines (tumor necrosis factor α [TNF-α], interleukin [IL]-1β, and IL-6) in colorectal cancer. It also inhibited the expression of proliferating cell nuclear antigen (PCNA) and B2 cell lymphoma (Bcl-2), through increased anti-Bcl-2-associated death (Bad) as well as the expression and activation of cleaved caspase-3 of the mitochondrial pro-apoptotic pathway. On the other hand, Martinez et al. [49] attributed the high antioxidant activity, detected for the EO hydroalcoholic extract by the 2,2-diphenyl-1-picrylhydrazyl (DPPH), the 2,2’-azinobis-3-ethylbenzothiazoline-6-sulfonic acid/Trolox Equivalent Antioxidant Capacity (ABTS/TEAC), the ferric reducing ability of plasma (FRAP) and the oxygen radical absorbance capacity (ORAC) assays, to its large content of total phenolic compounds (37.08 ± 8.56 g of gallic acid equivalent/100g). In a cell viability test, the same authors observed a decrease in A549 lung cancer cell viability, with cell cycle regulation due to cell increase in the G0/G1 diploid phases (2 n), but a reduction in the S (>2 n but <4 n) and G2/M (4 n) phases, in addition to a high increase in the apoptotic cells when compared to the untreated ones.

#### 3.1.3. Anticlastogenic Effect

The ability of EO extract to inhibit osteoclastogenesis and osteoclast activity was investigated by Brito et al. [51]. The 3-[4,5-dimethylthiazole-2-yl]-2,5-diphenyltetrazolium bromide (MTT), half maximal inhibitory concentration (IC_50_) and total protein assays showed that the EO extract concentration did not influence cell viability of monocyte macrophage cell line (RAW 264.7). However, its administration resulted in the inhibition of osteoclast differentiation and activity, possibly due to the modulation of cytokines produced by osteoclast precursor cells [51].

#### 3.1.4. Anticonvulsant Effect

Arrifano et al. [35] reported that EO juice can improve GABAergic neurotransmission, thus helping to treat seizures and epilepsies. Treatment of primary cultures of cortical neurons and astrocytes with EO (0–25%) resulted in an increase in binding of the agonist ([^3^H] flunitrazepam) and a decrease of the antagonist ([^3^H] TBOB) to their receptor binding sites on cortical neurons. Low concentrations still significantly inhibited GABA uptake, suggesting an accumulation of endogenous GABA in the synaptic cleft. Souza-Monteiro et al. [20] demonstrated the ability of EO juice to increase latencies to the first myoclonic jerk and first generalized tonic-clonic seizure, while reducing the total duration of tonic-clonic seizures caused by pentylenetetrazol administration, hence displaying an anticonvulsant response. It was also able to prevent lipid peroxidation in the cerebral cortex, highlighting an additional neuroprotective effect on humans against lipid peroxidation associated with seizures.

#### 3.1.5. Antidepressive and Anti-Aging Effects

EO juice was also tested for possible antidepressive and anti-aging effects [52]. Only 4 doses of juice were enough to prevent despair- and anhedonia-like behaviors, and changes observed in electromyographic measurements were comparable to those of imipramine. Tests by two-step quantitative reverse transcription polymerase chain reaction (qRT-PCR) highlighted an increase in the expression of telomerase reverse transcriptase (TERT mRNA) and an aging-associated enzyme whose level decreased in people with depression, revealing for the first time an anti-aging and neuroprotective action related to old age.

#### 3.1.6. Antidiabetic Effect

De Bem et al. [38] observed a positive antidiabetic effect when administering EO seed extracts. Dietary complications were induced in rats by a high-fat diet and streptozotocin; however, the reduction in glycemic indexes, insulin resistance, leptin and IL-6 levels, lipid profile and vascular dysfunction were evident in the treated group. When physical exercise was added to the treatment, it not only potentiated the reductions of glycemic indexes and TNF-α levels, but also increased the expressions of phosphorylated protein kinase B (pAKT), adiponectin in adipose tissue, and insulin receptor (IR) and phosphorylated adenosine monophosphate-activated protein kinase (pAMPK) in the skeletal muscle of type 2 diabetic rats.

#### 3.1.7. Antihypertensive Effect

The vasodilating effect on the mesenteric vascular layer of a rat pretreated with noradrenaline was evaluated. Rocha et al. [37] observed that the exposure to EO seed extract induced endothelium-dependent vasodilation, likely due to activation of the NO-cGMP pathway, which may involve the release of endothelium-derived hyperpolarizing factor (EDHF). According to Cordeiro et al. [53], EO seed extracts were able to attenuate spontaneously hypertensive episodes in rats due to high levels of protein carbonylation combined with low levels of nitrite, both attenuated in mesenteric arteries and heart homogenates. The up-regulation of nitric oxide synthase from endothelial tissue (eNOS) and superoxide dismutase 1 (SOD1) expression and increased SOD activity were also observed. EO also prevented endothelial dysfunctions in the aorta with increases in media thickness and media/lumen ratio and a decrease in the percentage of elastic fibers. These authors ascribed the antihypertensive activity to the antioxidant effects related to the high amounts of procyanidins and catechins in the extract along with the production of endothelial NO.

#### 3.1.8. Anti-Inflammatory Effect

As can be seen in Table 2, the anti-inflammatory effect of EO is one of its pharmacological activities most reported in the literature. Most in vitro studies report positive results of the ORAC assay and decreased ROS production even when the cell line used was exposed to lipopolysaccharides. Machado et al. [57] observed a reduction in the activation of murine RAW 264.7 macrophage line cells induced by the treatment with an EO hydroalcoholic extract, due to a decreased activation of nod-like receptor pyrin containing 3 (NLRP3). In another study, Poulose et al. [31], using BV-2 murine microglial cells, observed that the p38 mitogen-activated protein kinase (p38-MAPK) and the transcription factor NF-kB had their activities attenuated by EO, which regulated the production of Ca^2+^-independent nitric oxide synthase (iNOS) as well as the activation of cyclooxygenase 2 (COX -2). Zhou et al. [54] also reported that EO extract reduced the inflammation mediated by interleukin 8 (IL-8), tumor necrosis factor-α (TNF-α) and transforming growth factor-β (TGF-β) and reduced the alcohol-induced expression of nuclear factor NF -κB and CD68 in hepatocytes of Wistar rats. This study also reports a reduction in oxidative stress and liver damage.

#### 3.1.9. Antilipemic Effect

Martino et al. [39] claim that EO polyphenols reduce the accumulation of lipids in adipocyte cells by a reduction of the genetic expression of adipogenic transcription factors C/ebpα, C/ebpβ, Kruppel-like factor and sterol regulatory element-binding protein 1c, accompanied by a reduction in adipogenic genes, adipocyte fatty acid-binding protein 2, lipoprotein lipase, fatty acid transport proteins, fatty acid synthase, leptin, total plasminogen activator inhibitor, and by an increase in adiponectin level. These authors also reported an anti-inflammatory and antioxidant activity. In vivo studies demonstrated a hypocholesterolemic action of EO in rats that underwent diets rich in fat and cholesterol [60,61,62,63,64,65,66]. Feio et al. [65] observed that the consumption of EO juice improved the lipidic profile of adult male New Zealand white rabbits after 12 weeks of treatment, besides mitigating atherosclerosis. These effects were justified by an improvement of the balance of synthesis and absorption of sterols. In a clinical study by Pala et al. [66], a group of women who incorporated in their diet an amount of 200 g of lyophilized EO pulp for 4 weeks, showed reduced levels of ROS and oxidized-low density lipoproteins (ox-LDL) along with an increased activity of antioxidative paraoxonase 1. As far as the apolipoproteins involved in cholesterol metabolism are concerned, an improvement in the metabolism of Apo-I was observed, which implies an improvement in plasma high density lipoprotein (HDL) levels.

#### 3.1.10. Antimicrobial Effect

A study by Sprenger et al. [67] observed that hydroalcoholic extracts from EO seeds and fruit exerted an antimicrobial activity against strains of *Clostridium perfringens*, *Streptococcus aureus*, and *Pseudomonas aeruginosa*. Dias-Souza et al. [24], who tested a methanolic extract from EO pulp according to the serial dilution method, obtained positive results against *S. aureus* and its biofilm formation and observed a synergism when the extract was used in combination with antimicrobial drugs. For tests with EO extract, Borges et al. [68] used two strains of *Aspergillus fumigatus*, AFAR and AF4091, commonly found adhered to abiotic surfaces of medical-hospital material. Both the adhesion and the amount of fungal biomass, especially those of the AFAR strain, were remarkably reduced after treatment with EO.

#### 3.1.11. Antinociceptive Effect

A clinical study by Jensen et al. [69] demonstrated that the consumption of juice from EO fruits significantly improved amplitude and pain score data as well as the activities of daily living in patients. The antioxidant activity assessed by the novel cell-based antioxidant protection in erythrocytes (CAP-e) bioassay was improved over 12 weeks of treatment. Whereas tests with the C-reactive protein (CRP) inflammatory marker showed no significant difference, the lipid peroxidation was decreased. These results suggest an improvement in the physical well-being of patients with movement restrictions associated with pain caused by their daily load. Marinho et al. [42], who reported the first evidence of an antinociceptive effect of extracts from EO flowers and stalks, observed in rats a reduction of up to 50% in the total number of abdominal contortions, showing a peripheral effect of either extract in a dose of 30 mg/kg. Even though stalk extracts caused an increase in the rate of analgesia in the tail-removal model at doses of 10 and 30 mg/kg, no extract was able to change the analgesia index in the hot plate test, suggesting that there was no central action. Sudo et al. [70] used different methodologies to investigate the antinociceptive activity of EO seed extract (ASE) in an acute and chronic way on male Swiss mice and male Winstar rats, as well as the mechanisms underlying these effects. The hot plate test was used to evaluate antinociceptive agents that act centrally, but not peripherally. The treatment with ASE (30, 100, or 300 mg/kg/day) showed a dose dependent antinociceptive activity, as well as other methodologies such as the formalin-induced hind paw-licking test, the carrageenan-induced pain test and the acetic-acid writhing test. The study was not able to completely demonstrate the exact mechanism of action of the extract, suggesting the involvement of various pathophysiological systems.

#### 3.1.12. Antioxidant Effect

Hydroalcoholic extracts from EO seeds were used in antioxidant tests. The in vitro study on immortalized human umbilical vein cells (HUVEC) carried out by Soares et al. [72] proved that EO was able to prevent the deleterious effects caused by the H_2_O_2_-induced oxidative stress, besides positively modulating the signaling cascade of NRF2. An in vivo study showed that the same extract had a beneficial effect on the general framework of the cachectic syndrome caused in rats [73]. Carvalho et al. [74] demonstrated the antioxidant activity by the DPPH and ORAC assays of both a lyophilized EO pulp and an extract-containing gel in different concentrations (8%, 12%, 16%, and 20%).

#### 3.1.13. Antiplasmodial Effect

Ferreira et al. [76] tested three fractions obtained from the EO pulp, namely total phenolics, total anthocyanins and non-anthocyanidin phenolics. The first two fractions were inefficient in reducing parasitic DNA, while the third one showed moderate antiplasmodial activity in strains of *Plasmodium falciparum*. A murine model of infection was also used to investigate whether EO polyphenols could mitigate parasitemia in vivo, and *Plasmodium chabaudi*-infected mice were treated orally with the total phenolics fraction (10, 15, and 20 mg/kg) for 12 days. During parasitemia peak occurred after six and seven days, the 20 mg/kg dose reduced parasite growth by 89.4% and 77.3%, respectively, compared to the untreated control group, while the 15 mg/kg one did so by 81% and 62.2%, respectively.

#### 3.1.14. Antiproliferative Effect

Pozo-Insfran et al. [77] exposed HL-60 leukemic cells to different fractions rich in anthocyanins and other polyphenolic compounds known for their antioxidant activities. The biological response demonstrated a decrease in dose-dependent cell viability. Fractions containing non-hydrolyzed anthocyanins effectively suppressed HL-60 cell proliferation by inducing apoptosis via caspase-3 activation. Pacheco-Palencia et al. [29], in a comparative study on the proliferative action of polyphenolic fractions obtained from EO pulp and oil, noticed that both fractions caused a decrease in the viability of concentration-dependent human colon adenocarcinoma (HT-29) cells. The polyphenolic extract obtained from the oil proved to be twice as effective in all tested concentrations. When the same research-group tested monomeric and polymeric anthocyanins obtained from the fruit pulp on the same cell line, the former proved more effective [36].

#### 3.1.15. Antiprotozoal Effect

EO juice reduced the number of Leishmania promastigotes, caused morphological changes and increased ROS production [26]. The phenotypes of induced cell death were likely associated with apoptosis in promastigotes of *L. mazonensis* and *L. infantum* (= *L. chagasi*). The treatment against amastigotes incubated in isolated macrophages of peritonitis showed a reduction in the levels of cytokines of IL-17 family, which are involved in the pathogenic process, as well as a decrease in the number of intracellular amastigotes of the macrophages infected by both species. In addition, no cytotoxic effects were observed in infected macrophages, demonstrating leishmanicidal activity and safety to the host cell. Flavonoids present in the EO juice may have been responsible for the observed property [26].

#### 3.1.16. Cardioprotective Effect

Treatment performed with EO seed extract improved cardiac dysfunction and exercise intolerance in rats with induced myocardial infarction (MI) [79]. After treatment, the low systolic blood pressure (86.88 ± 4.62 mmHg) and high diastolic pressure (17.62 ± 1.21 mmHg) in animals with MI underwent inversion to values (130.00 ± 8.16 mmHg and 3.69 ± 2.69 mmHg, respectively) close to those of the control group. The distance covered by the treated group was 5.46-fold that of rats with MI, preventing cardiac hypertrophy, fibrosis, and dysfunction. A brief study showed a significant reduction in systolic blood pressure in healthy volunteers who had ingested EO gelatin capsules, but hemodynamic and electrocardiographic effects did not show any significant variation [80].

#### 3.1.17. Healing Effect

A study on cell proliferation showed increased migration of Hs68 human fibroblastic cells after administration of aqueous EO extract, which may play an important role in wound healing. EO increased the level of fibronectin mRNA expression and decreased that of matrix metalloproteinase MMP-1 mRNA expression. In vivo tests with macroscopic and histopathological observations showed a wound healing effect, indicating that EO is a potential healing agent [81]. The same research-group, treating an oral healing model with EO, observed effects on the healing of wounds in the oral mucosa consistent with previous histopathological observations [82].

#### 3.1.18. Cytotoxic effect

Cardiotoxicity studies showed that EO supplementation was able to prevent changes caused by doxorubicin, commonly used in chemotherapy [94]. The added EO diet improved fractional shortening of the left ventricle and increased the levels of enzymes associated with cardiac metabolism, such as β-hydroxyacyl-CoA dehydrogenase, phosphofructokinase, citrate synthase, complex II enzyme activities, and ATP synthase. On the other hand, the concentration of myocardial lipid hydroperoxide and the activity of the matrix metalloproteinase MMP-2 decreased when doxorubicin was used alone [34].

#### 3.1.19. Hepatoprotective Effect

Açaí seed extract reduced body weight gain, food intake, and glycemic, cholesterol, and triglyceride levels in the liver. Expressions of phosphorylated adenosine monophosphate activated protein kinase (pAMPK), phosphorylated acetyl-CoA carboxylase (pACC), acetyl-CoA carboxylase (ACC) and cholesterol excretion transporters [ATP-binding cassette, subfamily G transporter 5 (ABCG5) and ATP-binding cassette, subfamily G transporter 8 (ABCG8)] were increased, whereas those of lipogenic proteins [sterol-regulatory-element binding protein-1c (SREBP-1c) and 3-hydroxy-3-methylglutaryl CoA reductase (HMG-CoA reductase)] were reduced. These responses contributed to the reduction of obesity and hepatic steatosis. Antioxidant activity was also assessed, and there was an improvement in the activities of SOD, catalase and glutathione peroxidase [60]. De Freitas Carvalho et al. [84], who investigated non-alcoholic fatty liver disease, obtained answers in agreement with the study by De Oliveira et al. [60], when male mice of the C57BL/6 strain were treated with EO pulp extracts. The extract attenuated liver damage, inflammatory process and oxidative stress and modulated glutathione reductase, SOD and catalase, hence conferring a hepatoprotective effect ascribed to EO phenolic compounds. Both studies were cited earlier in the antilipemic effect subsection.

#### 3.1.20. Immune System Inhibition

Exposure of IgE-sensitized mouse primary cultured mast cells to EO pulp resulted in suppression of IgE-mediated degranulation and transcription of the cytokine genes from a cultured mast cell line originated from rat basophilic leukemia (RBL-2H3), selectively inhibited FcεRI signaling pathways and suppressed the FcεRI-mediated complementary signaling pathway. In general, EO inhibited IgE-mediated mast cell activation [85].

#### 3.1.21. Neuroprotective Effect

Exposure of rat pheochromocytoma cells (Ordway PC12) to human amyloid-protein 1–42 (Aβ_1–42_) or 25–35 (Aβ_25–35_) or to *tert*-butyl hydroperoxide decreased cell viability, whereas a pretreatment with EO extract only improved cell viability after exposure to Aβ_1–42_. EO extract and isolated metabolites such as (-)-epigallocatechin-3-gallate, cyaniding glucoside, cyaniding rutinoside caused loss of thioflavin T (ThT) fluorescence, with cyaniding glucoside also disrupting Aβ_1–42_ fibril and aggregate morphology [86]. A study by Machado et al. [87] suggested the use of EO as an alternative therapy for the treatment of bipolar disorder associated with mitochondrial dysfunction and cell oxidative stress. Exposure to EO extract increased enzyme activity of the mitochondrial complex I, amounts of proteins and, mainly, protein overexpression in mitochondrial complex I Q module subunits NDUFS7 and NDUFS8, and reduced levels of ROS and cellular lipid peroxidation.

#### 3.1.22. Lung Protective Effect

Polysaccharide fractions from EO fruit stimulated γδ T cell activity in peripheral blood mononuclear cells (PBMC) from humans, mice and cattle. High molecular weight polysaccharides isolated from crude EO were the most active in vitro activating myeloid T cells and γδ, while in vivo they induced the recruitment of myeloid cells and the production of IL-12, thus favoring a downstream Th-1 response able to alleviate asthma symptoms [88]. In a second study, the same polysaccharide fraction, when administered via nasal route in mice, a) protected animals against *Francisella tularensis* with survival rates of up to 80%, b) increased intracellular expression of interferon–gamma (IFN-γ) by natural killer (NK) cells in the lungs of infected animals, c) drastically reduced the number of *Burkholderia pseudomallei* cells in the lung, d) blocked the bacterial spread to the spleen and liver, and e) increased the IFN-γ response by NK and γδ T cells [89], thereby suggesting an agonistic action on the innate immune system.

#### 3.1.23. Renoprotective Effect

Exposure to EO seed extracts attenuated kidney damage in rats that had suffered diabetes induction, prevented its dysfunction, lowered serum levels of urea, creatinine and albumin, and reduced renal fibrosis due to the decreased expression of collagen IV and transforming growth factor beta 1 (TGF-β1), an early marker of fibrosis. Despite the contrast to the increase in pro-inflammatory biomarkers (IL-6, TNF-α and MCP-1) and in apoptosis (caspase-3), markers of oxidative damage [thiobarbituric acid reactive substances (TBARS), carbonyl and 8-isoprostane levels] were reduced [91]. On the other hand, there was an evident increase in the number of glomeruli and activity of antioxidant enzymes (SOD, catalase and GPx) as well as a reduction in inflammation and oxidative stress. A similar study carried out by Da Costa et al. [92] demonstrated that the treatment with EO seed extracts reduced kidney damage in rats with renovascular hypertension, likely due to antihypertensive and antioxidant effects.

#### 3.1.24. Hematopoietic Effect

A study by Shibuya et al. [93] showed a significant increase in the number of erythrocytes, hemoglobin and hematocrit in the C57BL/6NCrSlc mouse strain treated with a dose of EO fruit extract for 4 days. This erythropoietic effect was associated with renal hypoxia caused by EO. Tests showed an increase in the hematopoietic hormone and in the transient gene expression of the hematopoietic factor (EPO), expressed mainly in the kidney. There was also an increase in the expression of VEGFA, mediated by the transcription factor HIF induced by hypoxia, after exposure to EO. These authors suggested investigating other mechanisms of action for this EO function.

### 3.2. Pharmacological Applications of Euterpe oleracea Mart. Oil

#### 3.2.1. Antineoplasic Effect

Monge-Fuentes et al. [95] demonstrated an effective anticancer activity of a nanoemulsion formulated with EO oil against melanoma cell lines, leading to death by apoptosis/necrosis of 85% of line B16F10 cells in vitro and an 82% reduction of tumor volume in tumor-bearing C57BL/6 mice. These results suggest photodynamic therapy for the treatment of melanomas (Table 3).

#### 3.2.2. Anti-Inflammatory Effect

Favacho et al. [19] reported inhibitions in the formation of subcutaneous granulomatous tissue and ear edema, decreases in vascular permeability response and prostaglandin synthesis, and a reduction in up to 80.14% of migrating neutrophils in peritoneum inflammation, when compared to the control group.

#### 3.2.3. Antilipemic Effect

Sousa et al. [40], after inducing dyslipidemia in rats and treating them with EO, observed a reduction in the levels of triglycerides (TG), total cholesterol (TC), and direct low-density lipoprotein cholesterol (LDL-c), but an increase in that of HDL.

#### 3.2.4. Antimicrobial Effect

After *Pseudomonas aeruginosa* and *Staphylococcus aureus* growth in a medium composed of agar Muller–Hinton, disks containing 10 µg of EO seed oil were added. The oil-impregnated discs showed no inhibition halo after 24 and 48 h of contact with the medium containing *P. aeruginosa*, while that containing *S. aureus* induced an inhibition halo, even if significantly smaller than those induced by vancomycin and penicillin [23]. The minimum inhibitory concentration of the oil obtained from the pulp was determined against *Enterococcus faecalis*, *S. aureus*, *P. aeruginosa* and *Escherichia coli* strains by diffusion in broth. Oil inclusion complexes in (β) or (HP-β)-cyclodextrins showed a reduction in their minimum inhibitory concentration, increasing the oil’s antibacterial effect, which was related to the improvement of oil solubility and stability induced by complexation [25].

#### 3.2.5. Antinociceptive Effect

Favacho et al. [19] carried out an abdominal contortion test in rats that showed inhibition of up to 55.58% at a dose of 1500 mg/kg of oil. This effect was attributed to an action of peripheral origin and linked to the previously mentioned inhibition of prostaglandin biosynthesis.

#### 3.2.6. Cytotoxic Effect

An oral toxicity test using EO oil showed that doses of 30, 100 and 300 mg/kg caused changes in follicular morphology of thyroid cells, mainly marked by the loss of follicular cell lumen size, hypertrophy and unorganized growth. Meanwhile, hepatocytes underwent vacuolization and change from eosinophilic to basophilic characteristics. This suggests that morphological change caused a significant cytotoxic physiological change in both tissues [96]

#### 3.2.7. Genotoxic Effect

A genotoxicity study was carried out with 1% EO oil in Tween 80 at doses of 30, 100, and 300 mg/kg. The genotoxic property of EO oil was evaluated by comet assay, and samples of leukocytes obtained from peripheral blood, liver, bone marrow, and testicular cells did not show any indication of genotoxic effect [97].

## 4. Inventions Related to EO as an Alternative Medicine

Due to the above broad pharmacological arsenal, it is understandable that patentable inventions dealing with EO are developed for therapeutic purposes. In 2008, two patents were granted by Soares de Moura, researcher from the Federal University of Rio de Janeiro (Brazil) (Table 4). Patents were pertinent to the development of gelatin capsules and tablets obtained from lyophilized hydroalcoholic extracts of EO fruits and seeds, even though they point to different biological activities of the formulations.

The first patent filed, PI0418614-1 A2 [98], discloses obtaining said extracts and pharmaceutical forms for their application in pain treatment. The analgesic effect was determined from the antinociceptive response by the abdominal writhing test in mice. Abdominal writhing was induced intraperitoneally by acetic acid, and then the lyophilized extracts obtained by hydroalcoholic extraction from fruits were administered at doses of 1 and 3 mg/kg, while those from seeds at doses of 0.3, 1.0, 3.0, 10.0, and 30.0 mg/kg. All extracts were able to significantly reduce the number of abdominal contortions, and the antinociceptive response was improved as the concentration was increased. The patent suggests the use of these extracts for pain treatment as a general attempt, since the method is sensitive to antinociception in the central and peripheral nervous system, muscle relaxants, and sedatives [99,100].

The second patent, PI 0604281-3 A2 [101], reports the application of these same lyophilized extracts to treat and prevent vasospastic, ischemic diseases and hypertension. A vasodilation test was performed on mesenteric vessels of Wistar rats, cannulated and perfused with Krebs nourishing solution, after euthanasia laparotomy. After perfusion had been established, vasoconstriction with norepinephrine was induced, and then increasing doses of lyophilized extracts were administered, thereby obtaining a vasodilatory dose-response curve as a function of the decreased norepinephrine pressor effect. Approximately 2.58 and 1.21 µg of fruit and seed extracts, respectively, ensured 50% of the vasodilator effect observed. The experimental response suggests the application of these extracts to reduce vascular spasms, which are responsible for ischemic heart disease and ischemic peripheral vascular disease [102].

Examination of the selected patents revealed that several studies were still developed with hydroalcoholic extracts from EO fruits. In 2011 a third patent, WO 2011/1036448 A1, was issued by Soares de Moura [103], where he claimed the preparation of ointments or creams containing antioxidants from plants, especially those of the genus *Euterpe*, capable of accelerating the healing process (Table 4). After injuries to the epithelial tissue, inflammatory processes were triggered in response to the immune system and tissue healing. Such a mechanism stimulated the formation of ROS and reduced that of nitric oxide. When pro-oxidant responses exceed the potential of cellular antioxidant mechanisms, they may compromise cell viability and generation of secondary reactive species, rendering the healing response deficient [104].

The invention was based on scientific data obtained from international literature, which showed the presence of polyphenolic compounds in EO fruit and important antioxidant activity by preclinical assays [21,37,75,105]. In addition to the antioxidant activity, experiments also demonstrated the ability of these extracts to stimulate the expression of eNOS, which is responsible for the synthesis of nitric oxide [37] involved in wound repair.

Another invention has been developed with a similar purpose of the patent above. The invention, US 61/814791 [106], requested by Neocutis SA (Pully, Switzerland) and published in 2015, describes the addition of various plant extracts, quoting EO berries, to a preparation also containing vitamin C and E with therapeutic and preventive potential against skin damage (Table 4). Application of the EO extract was likely suggested by the high content of polyphenols in berries, which are responsible for a reduction of the oxidative stress caused by inflammatory, infectious, injury and tissue aging processes [17,21]. However, the addition of EO berry extracts to the above pharmaceutical formulations was only mentioned in the summary and claims with no additional information.

Nascimento, in 2018, patented (BR 102015017543-4 A2) the development of gelatin capsules and syrups for the treatment of breast cancer [107] (Table 4). The formulations were developed from hydroalcoholic extracts of EO and *Euterpe precatoria* seeds submitted to the freeze-drying process. The lyophilizates were applied to the MCF-7 human breast cancer cell line to investigate inhibition of cell proliferation and morphological changes. For this purpose, cells were seeded and incubated in 96-well plates under biochemical oxygen demand conditions and subjected to transmission electron microscopy. The results showed that the EO lyophilizates were cytotoxic at concentrations of 10 µg/mL and significantly reduced MCF-7 cell viability at concentrations of 20 and 40 µg/mL. Moreover, cells treated with 40 µg/mL of the freeze-dried extracts suffered severe morphological changes such as cytoplasmic retraction, vacuolization and apparent lysis with loss of cytoplasmic content. Accordingly, the invention patents the use of lyophilizates for chemotherapeutic purposes as described in claim 7.

Due to the EO fruit chemical composition, its application becomes desirable because of the high concentrations of metabolites responsible for significant antioxidant activity [30,31,36,39]. The patent BR 102017007451-0 A2 [108], published in 2018 (Table 4), discloses the formulation of tablets, capsules, granulates and controlled-release pharmaceutical preparations from ethanolic EO extracts. Plant extracts were subjected to quality control analysis and quantification of chemical constituents by thermal analysis, microscopic examination, spectroscopy in the infrared region and high-performance liquid chromatography (HPLC) coupled to mass detectors, while formulations were subjected to physicochemical tests as recommended by the V Brazilian Pharmacopoeia. Preclinical tests of acute toxicity [109] and determination of antioxidant activity by DPPH and ABTS•^+^ assays were performed to justify application.

The patent BR 102018005450 [110] discloses the development of, possibly lyophilized, powders obtained from açaí seed extracts and fractions thereof (Table 4). The invention has been developed to treat and prevent diseases or disorders individually or in conjunction with metabolic syndromes such as abdominal obesity, disorders of lipoprotein lipid metabolism, high levels of triglycerides, high uric acid levels, increased blood pressure, insulin resistance, glucose intolerance, increased fasting glucose and liver steatosis. Quantification of the extract and its fractions by HPLC confirmed the presence of procyanidin B2, epicatechin, rutin, catechin, vicenin 2, vitexin, chlorogenic acid, while in vitro artery relaxation assays evidenced effects ascribable to the presence of such compounds, especially procyanidin B2. These are the only trials reported in the patent, although the claim suggests broad pharmacological action.

The patent BR 10201807679332018 [111], unlike the others, shows the production of cycloamylases by encapsulating EO oil in cyclodextrins. The authors suggested that this system is a pharmacological alternative to increase the oil’s solubility and stability, thereby enabling the compound’s anti-inflammatory, antimicrobial and antioxidant effect. In this patent, no in vitro or in vivo analysis was shown, but the authors stressed the importance of using inclusion complexes with EO as a contribution to optimize the therapeutic arsenal. This invention arose from the wide knowledge and growing importance attributed to EO and its oil.

Recently, in 2019, the patent BR 102017013494-6 A2 [112] was published with the purpose of developing an antimicrobial phytotherapic from EO leaf extracts (Table 4). Even though the invention does not show the development of in vitro or preclinical trials, it proposes the development of experiments to support the suggested biological activity.

EO extracts and oils are commonly consumed by the population because of their biological properties, especially in the treatment of inflammation. The number of studies conducted with fruits, leaves, seeds, or oil of EO shows the importance and interest of researchers to this plant, especially due to its chemical composition and biological activities discovered in scientific experiments. However, it is understood as contradictory the number of patents located, whose purpose suggests their pharmacological application.

**Figure 4:**
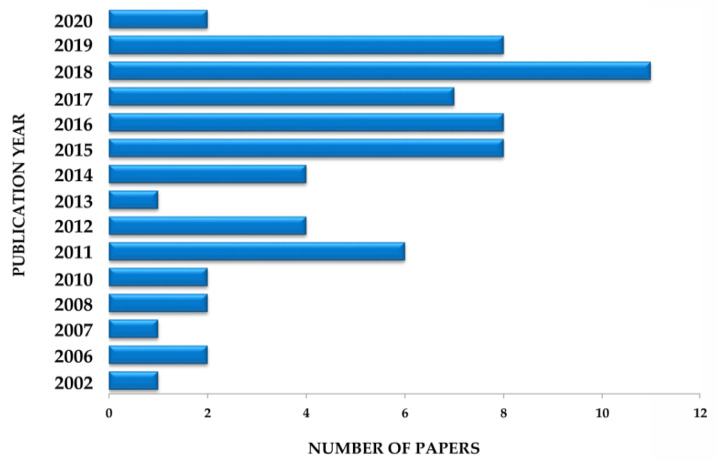
Number of papers reporting the pharmacological activities of *Euterpe oleracea* Mart. for the treatment and prevention of general diseases.

When searching for publications on EO biological properties, only one scientific article was found in 1984 by Plotkin and Balick [15], in which the authors described the use of EO oil as antidiarrheal in folk medicine. Until 2002, no other article related to its pharmacological application was found in literature. In the past ten years there has been an increasing interest in the discovery of possible biological activities of EO. Six publications were recorded in 2011 and eight in 2015 and 2016. However, the largest record was in 2018, with 11 publications (Figure 4).

**Figure 5:**
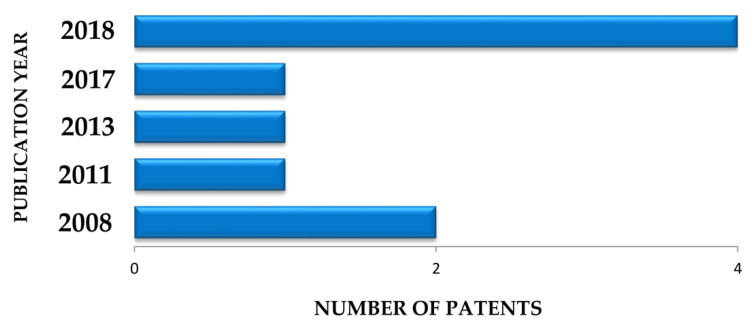
Number of patents reporting pharmacological activities of *Euterpe oleracea* Mart.

After a detailed search of patents and their submission to the inclusion and exclusion criteria of this study, only nine patents suggested pharmacological EO application. A second point analyzed was the geographical distribution of patents. Moreover, 88.9% are Brazilian and 11.1% belong to the United States. The high percentage of Brazilian patents may be justified by the origin of EO, which is well distributed in the states of Pará, Maranhão, Amazônia, and Amapá in the north of Brazil [26,113]. The registration of the selected patents began in 2008 and the largest number of publications was in 2018 (Figure 5).

The inventions suggest the application of various EO fruit, seed and leaf extracts to treat bacterial and fungal infections, treat and prevent skin damage, accelerate healing, antioxidant activity, antinociceptive action to pain, chemotherapeutic and chemo preventive action against breast cancer, treat and prevent vasospastic and ischemic diseases, high blood pressure, and metabolic syndromes in general.

In 50% of the patents analyzed, preclinical trials of the formulations were done, while the other half only pointed to the development and pharmacological application of products based on previous studies reported in the international literature or suggested evidential experimental development. For these, the inventors recognize EO fruit composition and link it to possible biological activities. An example is the invention of an EO fruit extract ointment [103], in which the identification of polyphenolic compounds, well described in the literature, directly influenced the development of this patent. Therefore, if there is no defined mechanism of action and clinical evidence, subjectivity is generated in the content of such patents. In vitro and in vivo assays are primordial and necessary for accreditation of the invention.

In the same context, the patent filed by Neocutis S.A. [106] that pointed out a formulation containing vitamins C and E as well as an antioxidant compound, included EO berries among the possible natural extract of choice for the composition. EO was likely selected because of its contents of phenolic compounds, anthocyanins and phytosteroids, which give the fruit antioxidant and anti-inflammatory activities [31,36]. Nevertheless, the invention does not show any pharmacological effect or synergistic activity among the components of the formulation, raising questions about its efficiency.

Inventions corroborated by preclinical studies generate high expectations among patients, as is the case of studies carried out against breast cancer cells, which have shown chemotherapeutic activity of EO seed extracts [107]. The invention of tablets, capsules, and granules obtained from fruit extracts was the only one that evidenced physicochemical, quality control, efficacy, and safety tests throughout the development of the formulation [108], becoming extremely relevant for product accreditation. Importantly, no patent mentions clinical trials.

Considering that physicochemical, pre-clinical and clinical tests must be carried out in all stages of the development of a new medicine, it is evident that more accurate studies are needed on pharmaceutical formulations.

## 5. Survey Methodology and Criteria

This review provided a survey of the papers and patents related to the therapeutic and preventive activity of EO and the advances in its pharmacological application. For this purpose, a search was performed to identify articles in the scientific literature, while specialized databases were consulted for patents such as the National Institute of Industrial Property (INPI), Latin American Patent Bank (Latipat), European Patent Office (ESPACENET), World Intellectual Property Organization (WIPO) and United States Patent and Trademark Office (USPTO). For such a search we used the terms “*Euterpe oleracea*” or “açaí” appearing in the title or abstract. To prepare Table 2, we considered articles that suggested therapeutic and preventive activities for pharmacological purposes, while excluding those that only addressed biological activities without pharmacological indication, or simply suggested EO as a source of nutraceuticals or cosmetics, and review articles. Likewise, only patents reporting EO pharmacological and preventive activities were taken into consideration. The International Patent Classification (IPC), section A, related to human needs, and subclass A61K, preparations for medical, dental or hygienic purposes, were adopted. For both searches, there was no restriction in the years of publication.

**Figure 6:**
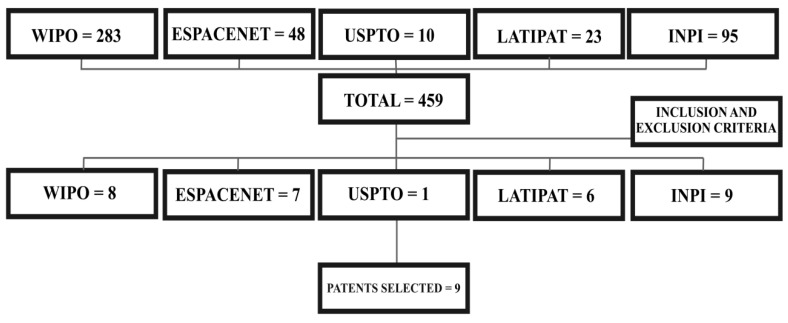
Selection of patents concerning pharmaceutical formulations of *Euterpe oleracea* Mart. for the treatment and prevention of general diseases.

The search revealed a total of 459 patents. Patents related to cosmetic, food and hygiene applications, even those conforming to the previously mentioned classification, have been excluded. Only some patents presented preclinical trials showing EO biological activity, others only suggested its pharmacological application, but all were considered in the present study. Also, as an inclusion criterium, only patents written in English and Spanish were selected. Thus, after applying the inclusion and exclusion criteria and withdrawal of multiple patents, eight patents were selected and analyzed according to the objective of the study (Figure 6).

## 6. Conclusions and Future Perspectives

*Euterpe oleracea* (EO) is a plant known all over the world especially for its biological activities, whose chemical composition varies according to the part considered. Extracts from the fruit, roots, and leaves are mainly composed of phenolic metabolites, while EO oil is basically composed of fatty acids. Therefore, they are considered abundant sources of bioactive extracts capable of maintaining health.

Several in vitro and in vivo tests have demonstrated a direct relationship between EO chemical composition and numerous pharmacological activities such as anti-inflammatory, antioxidant, antimicrobial, antinociceptive, anticancer, anti-atherogenic and healing activities, which may be usefully exploited to treat metabolic syndromes and protect the lungs, kidneys, liver, heart, and nervous system. As for the analyzed inventions, they showed the use of EO in the treatment of infections by bacteria and fungi, the treatment and prevention of skin damage, breast cancer, and metabolic syndrome and the acceleration of healing, besides its antinociceptive and antioxidant properties. The scientific literature and patents concerning EO suggest a recent and, at the same time, growing interest in this plant, with a set of actions pointing to the development of promising new drugs for clinical use. However, very few studies and no patents have prioritized clinical trials, suggesting that more efforts should be made to clarify the effectiveness and safety of promising crude extracts, as well as isolated components of this plant for the subsequent consolidation of a pharmaceutical product.

## Figures and Tables

**Table 1:** Chemical profile of different parts of *Euterpe oleracea* Mart. with pharmacological application.

Plant Part	Identified	References
	vanillic acid	
	syringic acid	
	*p*-hydroxybenzoic acid	
Pulp	protocatechuic acid	[29,30,31]
	ferulic acid	
	quercetin	
	(+)-catechin	
	cyanidin-3-glucoside	
	cyanidin-3-rutinoside	
	oleic acid (18:1)	
	linoleic acid (18:2)	
	palmitic acid (16:0)	
	palmitoleic acid (16:1)	
	myristic acid (14:0)	
	lauric acid (12:0)	
Oil	protocatechuic acid	[25,29]
	*p*-hydroxybenzoic acid	
	catechin	
	vanillic acid	
	syringic acid	
	ferulic acid	
	procyanidin dimers	
	procyanidin trimers	
	3-caffeoylquinic acid	
	4-caffeoylquinic acid	
	5-caffeoylquinic acid	
	6,8-di-C-hexosyl apigenin	
Leaf	6,8-di-C-hexosyl apigenin sulfate	[41]
	6-C-hexosyl-8-C-pentosyl apigenin isomers	
	6-C-pentosyl-8-C-hexosyl apigenin isomer	
	8-C-glucosyl luteolin	
	6-C-glucosyl luteolin	
	6-C-glucosyl apigenin	
	3-caffeoylquinic acid	
	4-caffeoylquinic acid	
Root	5-caffeoylquinic acid	[41]
	4-caffeoylshikimic acid	
	5-caffeoylshikimic acid	

**Table 2:** In vitro and in vivo studies reporting the biological activities of different parts of *Euterpe oleracea* Mart.

Model	Plant part	Assay/Dose	Results	Ref.
***Pro-apoptotic activity***				
In vitro method	Polyphenolic EO fraction	Analyses were conducted at concentrations of 5–20 mg/L.	Inhibition of HT-29 and SW-480 colon cancer cells. Absence of toxicity to CCD-18Co cells. Reduction of ROS induced by H_2_O_2_.	[45]
***Antineoplasic activity***				
In vivo method	Hydroalcoholic EO fruit extract	8-Week-old female Winstar rats were treated with 200 mg/kg/day of lyophilized EO fruit extract for 16 weeks.	Decrease in the number of inflammatory cells and positive cells for macrophages in breast tumors. Reduction in immunostaining of VEGF, VEGFR-2 and COX-2. Lower concentrations of PGE2, VEGF and IL-10 in comparison with the control group.	[22]
In vivo method	Lyophilized EO berries	4-Week-old male ICR mice received 2.5% and 50% açaí-containing diets for 14 weeks starting 1 week after colitis induction.	Reductions of the incidence of adenoma and cancer, MPO, TNF-α, IL-1β and IL-6 in colorectal cancer. Inhibition of PCNA and Bcl-2 expression and increased BAD and cleaved-caspase-3 expression.	[46]
In vivo method	Lyophilized EO pulp	Male Winstar rats received a basal diet supplemented with 5.0% and 7.5% of lyophilized açaí pulp for 22 weeks.	Reduction in the motility of colon carcinoma cells of the RKO line. Reduction in the total number of aberrant crypt foci, tumor cells, proliferation, and incidence of tumors with high-grade dysplasia. Increased gene expression of negative regulators of cell proliferation such as Dlc1 and Akt3.	[47]
In vivo method	Lyophilized EO pulp	4-Week-old male Swiss albino mice received 2.5% and 5.0% of lyophilized açaí pulp as an additive to their low-fat diets for 4 weeks.	Reduction of damage to DNA from peripheral blood cells and reduction of multiplication of crypts and preneoplasic lesions of colon cancer. Total glutathione increase.	[48]
In vitro method	Hydroalcoholic EO seed extract	1.25-200 µg/mL doses of the lyophilized extract were used for cell viability assay and 50 and 100 µg/mL for cell cycle performance and apoptosis.	High antioxidant activity by DPPH, ABTS, FRAP and ORAC. Decreased cell viability of lung cancer cell line (A549). Regulation of cell cycle, prevention of cell growth and increase in apoptotic cells.	[49]
In vitro method	Hydroalcoholic extract of EO seeds and peel	The extract was tested at concentrations of 10, 20 and 40 μg/mL in the MTT viability, Nuclear staining and Caspase-Glo^®^ 3/7 luminescent assays.	The MCF-7 breast cancer line was the only one that responded to treatment with EO. Significant reduction in cell viability, and cell morphological characteristics altered by the appearance of autophagic vacuoles.	[50]
***Anticlastogenic activity***				
In vitro method	Dry EO pulp extract	The extract was tested at concentrations of 25, 50 or 100 μg/mL in the osteoclast differentiation, cell proliferation, hydroxyl apatite resorption and cytokine assays.	There was no evidence of toxicity to RAW 264.7 cells. Decreased secretion of IL-1α, IL-6 and TNF-α, but increased secretion of IL-3, IL-4, IL-13 and gamma interferon.	[51]
***Anticonvulsant activity***				
In vivo method	EO juice	Male Swiss rats were treated (10 μL/g body weight) with açaí juice by gavage for 4 days.	Delay of the first tonic–clonic seizure and decrease in its total duration. Prevention of total lipid peroxidation in the cerebral cortex.	[20]
In vitro method	Clarified EO juice	The EO juice was tested in neurons and astrocytes at concentrations of 0-50% to a final volume of 250 mL in HBSS	Increased binding to the agonist and decreased binding to the antagonist in cortical neurons. GABA uptake in the synaptic cleft points to an accumulation of endogenous GABA in the synaptic cleft.	[35]
***Antidepressive activity***				
In vitro and in vivo methods	Clarified EO juice	Male Swiss rats were treated (10 μL/g body weight) with açaí juice by gavage for 4 days.	Prevention of depressive behavior and changes in electromyography. Increased expression of TERT mRNA. Prevention of lipid peroxidation. Reduction of nitric oxide levels.	[52]
***Antidiabetic activity***				
In vivo method	Hydroalcoholic EO seed extract	The extract was administered by gavage (200/kg/day) to male Winstar rats for 4 weeks. Animals also exercised on a treadmill (30 min/day; 5 days/week).	Activation of the insulin signaling pathway in muscle and adipose tissue, increased levels of GLP-1 and anti-inflammatory action. Physical training enhanced the glucose-lowering effect, activating the phosphorylated adenosine monophosphate-activated protein kinase pathway and increasing the expression of insulin receptor.	[38]
***Antihypertensive activity***				
In vivo method	Aqueous commercialized extract;Hydroalcoholic EO peel and seed extracts	Concentrations in the range 0.3–100 μg/mL were tested in the mesenteric vascular bed of male Winstar rats.	Vasodilation by activation of the cyclic guanosine oxide-nitric-monophosphate pathway and possibly by the release of the endothelium-derived hyperpolarizing factor.	[37]
In vivo method	Hydroalcoholic EO seed extract	A dose of 200 mg/kg/day was tested in spontaneously hypertensive Winstar rats (50 days old) for 70 days.	Attenuation of protein carbonylation and nitrite levels. Up-regulation of eNOS and SOD1 expression. Increased activity of SOD. Prevention of the increase in media thickness and media:lumen ratio and decrease of elastic fibers.	[53]
***Anti-inflammatory activity***				
In vivo method	EO fresh pulp	Pure EO pulp (1 mL/100g) was administered to Winstar rats by gavage for 8 weeks.	EO intake alleviated chronic alcoholic liver injury in rats by attenuating oxidative stress and inflammatory response.	[54]
In vitro method	Extract from freeze-dried EO pulp	Isolated compounds were used in antioxidant and anti-inflammatory assays.	Five flavonoids were isolated and structurally identified. ORAC values varied significantly based on their chemical structures. Velutin strongly inhibited SEAP secretion in RAW-blue cells induced by LPS or ox-LDL, suggesting anti-inflammatory effects.	[55]
In vivo method	Hydroalcoholic EO seed extract	8-Week-old male C57BL/6 mice were exposed to the smoke of 6 cigarettes for 5 days. Animals were treated with 300 mg/kg of the extract.	The group exposed to cigarette smoke exhibited lung morphology like that of the control group. The numbers of neutrophils and macrophages were lower than those not treated with EO. Reduction of pulmonary inflammation and oxidative stress markers.	[56]
In vitro method	Freeze-dried hydroalcoholic EO extract	Macrophages (RAW 264.7) were treated with different concentrations of the extract (0.001–1000 μg/mL) for 24 h at 37°C in a humid environment with 5% CO_2_.	The extract was able to reduce macrophage activation and proliferation through cell cycle arrest due to reduced activation of NLRP3 in response to the recovery of oxidative metabolism.	[57]
In vitro method	Polyphenolic compounds from EO juice	Polyphenolics were diluted to known concentration of total polyphenolics and normalized to contain a maximum concentration of 0.1% in DMSO (water: DMSO, 60:40).	The EO polyphenolic extract [1–5 mg gallic acid equivalent (GAE) L^-1^] had a protective effect against ROS production in human colon myofibroblastic CCD-18Co cells with and without LPS challenge.	[58]
In vitro method	EO fruit pulp fractions	The tested extract concentrations ranged from 50 to 1000 μg/mL for the methanol, ethyl acetate and acetone fractions, and from 10 to 250 μg/mL for the ethanol fraction.	LPS-mediated upregulation of p38-MAPK and NF-κB was attenuated by EO pulp fractions, which in turn down-regulated iNOS and COX-2 in BV-2 microglial cells.	[31]
In vivo method	Hydroalcoholic EO seed extract	Female Sprague-Dawley rats were treated with 200 mg/kg of extract dissolved in saline, by gastric tube for 30 days.	The EO extract effectively suppressed the establishment and growth of endometriotic lesions.	[59]
***Antilipemic activity***				
In vivo method	Hydroalcoholic EO seed extract	C57BL/6 male mice underwent a high fat (60%) diet. They also received 300 mg/kg^/^day of the extract by gavage for 12 weeks.	Mice fed with both high fat diet and EO seed extract showed a decreased food intake and body mass gain and had improved adiposity and hepatic steatosis.	[60]
In vitro method	Polyphenolic fraction from frozen pulp	Concentrations of EO polyphenols ranging from 2.5 to 10 μg GAE/mL were tested in mouse preadipocytes/fibroblast cells.	Reduced intracellular lipids accumulation during adipocyte differentiation in a dose-dependent manner and decrease in the expression of pro-inflammatory cytokines with and without TNFα challenge.	[39]
In vivo method	Ethanolic EO seed extract	N/D	Reduced proliferation of 3T3-L1 adipocytes. Inhibited proliferation of pre-adipocytes through transcription factors and adipogenic proteins such as PPARɣ, SREBP-1 and FAZ, suppressing lipid accumulation.	[61]
In vivo method	Pasteurized EO pulp	Two groups of female Fischer rats (standard diet + EO and hypercholesterolemic diet + EO) were supplemented with 2% of EO pulp for 6 weeks.	EO addition to diet had a hypocholesterolemic effect, reducing total and non-HDL cholesterol levels.	[62]
In vivo method	EO seed flour (ASF)	3-Month-old male C57BL/6 mice received a high fat diet of 150 g/kg and 300 g/kg ASF daily for 12 weeks.	ASF treated groups showed lower triglyceride accumulation in hepatocytes compared to groups receiving just high fat diet. ASF consumption had a positive effect on liver steatosis.	[63]
In vivo method	Pasteurized EO pulp	Female Fischer rats were divided into groups with different diets, one of which receiving a standard AIN-93M diet and 2% EO and the other a hypercholesterolemic diet supplemented with 2% of EO daily for 6 weeks.	EO had a hypocholesterolemic effect on dietary-induced hypercholesterolemia through an increase in the expression of ABCGs and LDL-R genes.	[64]
In vivo method	EO pulp juice	Adult male New Zealand white rabbits were fed a regular diet plus 0.5% cholesterol for 12 weeks.	Consumption of EO extract markedly improved the lipid profile and attenuated atherosclerosis. These effects were related in part to a better balance in the synthesis and absorption of sterols.	[65]
Clinical study	EO pulp	EO pulp was provided in 200 g portions and consumed at leisure daily for 4 weeks.	EO consumption decreased ROS, ox-LDL and malondialdehyde while increasing antioxidative paraoxonase 1 activity. The increase in apolipoprotein A-I and cholesteryl ester transfer to HDL after the EO intake period suggested improved metabolism of this lipoprotein. EO also proved favorable for plasma HDL metabolism and antioxidant defense.	[66]
***Antimicrobial activity***				
In vitro method	Methanolic EO pulp extract	The extract was serially diluted to concentrations ranging from 1 mg/mL to 7.8 μg/ mL.	The methanolic extract was effective against cells and biofilms of *S. aureus*. Combinations of the methanolic extract and antimicrobial drugs resulted in statistically significant synergism.	[24]
In vitro method	Hydroalcoholic extract from EO leaves, fruits, and seeds	Using the serial dilution method, eight different concentrations (10–2.560 μg/mL) of plant extract were obtained and tested for antimicrobial activity.	The extracts showed antimicrobial activity against *Clostridium perfringens, Streptococcus aureus* and *Pseudomonas aeruginosa*, while none of them had any effect against *Escherichia coli.*	[67]
In vitro method	Hydroalcoholic EO pulp extract	Extracts with concentrations of 7.8, 15.6, 31.2, 62.5, 125, 250, 500, 1000 μg/mL were tested.	Two strains of *Aspergillus fumigatus,* AFAR and AF4091, showed poor adhesion. Extracts inhibited AFAR more than AF4091 strain growth.	[68]
***Antinociceptive activity***				
In vivo method	Ethanolic extract of EO flowers and stalks	Doses of 1, 10 and 30 mg/kg were administered.	Both extracts caused a 50% reduction in the total number of abdominal contortions. Increased rate of analgesia in the tail-removal model by stalks extract. Absence of hot plate analgesia for both extracts.	[42]
Clinical study	EO fruit and berry juice	Participants consumed 120 mL of EO fruit juice daily for 12 weeks.	Significant reduction of pain, better scores of amplitude and pain associated with activities of daily living in patients.	[69]
In vivo method	Hydroalcoholic EO seed extract (ASE)	30, 100 or 30 mg/kg ASE samples were administered to male Swiss mice and male Winstar rats.	Reduction of nociception to acute/inflammatory pain, including thermal hyperalgesia, acetic acid-induced contortion and carrageenan-induced thermal hyperalgesia. Reduction of neurogenic and inflammatory phases after intraplantar injection of prevention of chronic pain in a rat spinal nerve attachment model.	[70]
***Antioxidant activity***				
In vitro method	Fractions from the mesocarp/epicarp and endocarp of EO fruit	Each sample extract was dissolved in 50% ethanol up to a concentration of 2 mg/mL.	In all antioxidant assays, including Hydrophilic (H-) and L-ORACFL assays, but except the ABTS radical quenching one, mesocarp/epicarp extracts showed stronger activity.	[30]
In vitro method	Compounds from powdered flakes of the fruit pulp and fractions	10 µL of test samples in 25% DMSO solution were used.	Nine lignans and 2,6-dimethoxy-1,4-benzoquinone exhibited potent antioxidant activities by the Hydroxyl Radical Scavenging Activity assay, and 7 lignans by the DPPH assay.	[71]
In vitro method	Hydroalcoholic EO seed extract	Immortalized human umbilical vein endothelial cells (HUVEC) were treated with different concentrations of EO seed extract (0.1 – 100 μg/mL).	The EO seed extract was able to prevent the deleterious effects of H_2_O_2_ induced oxidative stress in HUVEC and positively modulated the antioxidant transcription factor (Nrf2) signaling pathway.	[72]
In vivo method	Hydroalcoholic EO seed extract.	Winstar rats were treated with 100 and 200 mg/mL extract by gavage for 14 days.	The EO seed extract showed no beneficial effect on the general framework of the cachectic syndrome in lab rats.	[73]
In vitro method	Lyophilized EO pulp	Gels were formulated with concentrations of 8, 12, 16 and 20% lyophilized EO pulp and sugars.	The gels with the highest concentration of freeze-dried EO pulp showed higher antioxidant activity.	[74]
In vitro method	Freeze-dried EO fruit pulp/skin powder.	For the neutrophils assay, EO powder was added to phosphate-buffered saline solution. For the COX assay, EO powder was extracted with 50% acetone and tested directly.	Freeze-dried EO showed a positive response as a COX-1 and COX-2 inhibitor. This study also proved that antioxidants in freeze-dried EO are able to enter human cells in a fully functional form in vitro.	[75]
***Antiplasmodial activity***				
In vitro and in vivo methods	Polyphenolic fractions from EO pulp	Fractions: Total phenolics (1), non-anthocyanin phenolics (2) and total anthocyanins (3). For the in vitro study 1.0 to 20.0 mg/L concentrations were used, while for the in vivo mice infected with two *Plasmodium chabaudi* strains were treated with fraction 1 at doses of 10, 15 and 20 mg/kg/day for 12 days.	None of the doses of 1 and 2 reduced the DNA content of either parasite strain tested. Fraction 2 showed moderate antiplasmodial activity in both strains, starting at 10.0 mg/L. During parasitemia peak in the in vivo assay, all concentrations of fraction 1 showed a decrease in parasite growth.	[76]
***Antiproliferative activity***				
In vitro method	Polyphenolic-enriched fractions from EO pulp and oil	Polyphenolic isolates were sterile filtered, normalized to a final concentration of 0.1% in DMSO and tested on HT-29 colon cancer cells.	Both polyphenolic extracts caused a significant decrease in total HT-29 cell number in a concentration-dependent manner. However, the oil polyphenols extract was more than twice as effective at all dilutions.	[29]
In vitro method	Monomeric and polymeric anthocyanin fractions from EO pulp	50 and 500 µg of cyanidin-3-glucoside equivalent/mL of anthocyanin fractions were diluted in HBSS and tested on HT-29 colon cancer cells.	Both fractions and their mixtures decreased total cell numbers. The monomeric anthocyanin fractions (5-20 µg/mL) were more effective in reducing HT-29 cell proliferation.	[36]
In vitro method	EO pulp fractions	Nine fractions were obtained based on the solubility and affinity characteristics of the EO phytochemicals.	HL-60 leukemia cells showed a dose-dependent decrease in viability after 24 h-exposition to all EO fractions, with exception of the lipophilic and C18 non retained fractions (II and VI, respectively). Fractions I, III and V strongly suppressed HL-60 proliferation through apoptosis induction by caspase-3 activation.	[77]
In vitro method	Anthocyanin-rich EO pulp extract	The anthocyanin-rich extract was applied to cell cultures of C-6 rat brain microglia cells and MDA-468 breast cancer cells at concentrations of 50, 100 and 200 µg/mL.	The treatment suppressed proliferation of C-6 rat brain glioma cells significantly. However, the growth of MDA-468 breast cancer cells was not affected.	[78]
***Antiprotozoal activity***				
In vitro and in vivo method	Clarified EO juice	EO juice acquired from Amazon Dreams (Belém, PA, Brazil). Concentrations: 1: 12.5, 1:25 and 1:50 (v: v, EO and DMEM or RPMI medium culture).	Reduction in the number of *Leishmania* promastigotes and amastigotes. Reduction in cytokine levels of IL-17. Absence of cytotoxicity to macrophages infected by amastigotes	[26]
***Cardioprotective activity***				
In vivo method	Hydroalcoholic extract of EO seeds	Male Winstar rats were treated with 100 mg/kg/day of the extract for 4 weeks.	Positive modulation in systolic and diastolic blood pressure. Better distance covered when compared to the group with induced myocardial infarction.	[79]
Clinical study	EO fruit	Healthy individuals received gel capsule containing 500 mg of açaí provided by Nature’s Bounty Inc. (Bohemia, NY, USA).	Reduction of systolic blood pressure. Absence of variations in hemodynamic and electrocardiographic effects.	[80]
***Healing***				
In vitro and in vivo methods	EO aqueous extract	Concentrations of 0, 0.1, 0.3 and 1 mg/mL were tested in vitro, and a dose of 200 μL/wound/day for 18 days was tested in male Sprague-Dawley rats.	Increased expression of fibronectin mRNA. Decreased mRNA expression of MMP-1. Macroscopic and histopathological observations demonstrated healing.	[81]
In vivo method	EO aqueous extract	6-Week-old male Sprague-Dawley rats had 50 μL of the extract applied to the wound area once a day for 6 days.	Macroscopic and histopathological observations demonstrated healing. Significantly high antioxidant effects in the Electron donating ability and ABTS assays, although they were slightly lower than in the control group. Low SOD value.	[82]
***Cytotoxic activity***				
In vivo method	Commercialized EO pulp	A mixture containing 5% EO was tested in cytotoxicity assays.	Improvement of fractional shortening of the left ventricle. Increase in β-hydroxyacyl-CoA dehydrogenase, phosphofructokinase, citrate synthase, complex enzyme II and ATP synthase activities. Decrease in myocardial lipid hydroperoxide and MMP-1 activities occurring in doxorubicin cardiotoxicity.	[34]
In vitro method	Fractions of hydroalcoholic EO seed extract after liquid-liquid partition	Chloroform (CF) and hexane (HF) fractions were diluted in DMSO, while the ethyl acetate fraction (EAF) was diluted in Milli-Q water. MCF-7 cells were treated with HF, CF and EAF at concentrations of 10, 20, 40 and 60 µg/mL.	HF, CF and EAF promoted cell viability reduction. However, EAF was significantly more cytotoxic when compared to HF and CF after 48 h, hence indicating antineoplastic potential. Cell death occurred by necrosis, not apoptosis	[83]
***Hepatoprotective activity***				
In vivo method	Hydroalcoholic EO seed extract	High-fat diet mice were treated with 300 mg/kg/day.	Reduction in body weight gain and food intake. Reduction of glycemic indexes, cholesterol and triglycerides in the liver. Reduction in the expression of SREBP-1c and HMG-CoA reductase. Increased expression of pAMPK, pACC, ACC, ABCG5 and ABCG8.	[60]
In vivo method	Marketed EO seed flour	Diet was prepared containing 15 or 30% of commercially available EO seed flour (Prag Soluções, Jaú, SP, Brazil). Diet was administered for 12 weeks to 3-months-old male C57BL/6 mice.	Reduction of lipid, glycemic indexes of insulin, leptin and lipogenesis. Reduced expression of SREBP-1c and HMG-CoA reductase. Increased expression of pAMPK, pACC, ACC, ABCG5/8.	[63]
In vitro and in vivo methods	Commercialized EO pulp	Doses tested in transit: 0, 12.5, 25, 50, 100, 200 and 400 mg/mL; Doses tested in steatotic mice: 3 g/kg/day for 6 weeks.	Inhibition of ROS and absence of cytotoxicity in liver carcinoma cells. Reduction of alanine aminotransferase, number of inflammatory cells, serum TNFα, lipid peroxidation and protein carbonylation.	[84]
***Immune system inhibition***				
In vitro method	EO pulp fruit	EO pulp was filtered and added to the cell culture medium at 1/30 or 1/60 dilution.	Reduction of antigen-induced degranulation of mouse primary cultured mast cells. Inhibition of FcεRI signaling pathways and suppression of FcεRI-mediated complementary signaling pathway.	[85]
***Neuroprotective activity***				
In vitro method	EO extract and isolated fractions	Concentrations in the range 0.5 - 50 μg/mL were tested.	Better viability of rat pheochromocytoma cells (PC12). Loss of ThT fluorescence. Disrupted human amyloid-protein (Aβ1–42) fibril and aggregate morphology.	[86]
In vitro method	Hydroalcoholic EO fruit extract	SH-SY5Y cells were exposed to 5 μg/mL of açaí extract.	Increased enzyme activity of the mitochondrial complex I, amount of proteins and overexpression of the mitochondrial complex I Q module subunits NDUFS7 and NDUFS8. Decreased levels of ROS in cells and lipid peroxidation.	[87]
***Lung protective activity***				
In vitro and in vivo methods	EO fruit pulp; polysaccharides isolated from EO fruit powder	TLR42/2 and TCRa2/2 mice (both on the C57BL/6 background) were treated with 5 μg to 500 μg of polysaccharides. Human monocyte-macrophage MonoMac-6 cells were also used for in vitro assays.	Polysaccharide fractions obtained from EO stimulated the activity of T γδ lymphocytes in cultures. Isolated fractions of polysaccharides with high molecular weight activated myeloid T cells and γδ in vitro and induced myeloid cell recruitment and IL-12 production in vivo.	[88]
In vitro and in vivo methods	Polysaccharides isolated from EO fruit powder	RAW264.7 cells were treated with varying doses of the isolated polysaccharides. The mice were treated with 1000 μg of polysaccharides before infection.	Isolated polysaccharide fraction reduced the replication of *Francisella tularensis* and *Burkholderia pseudomallei* in the lung of EO infected mice. Increased response of IFN-γ by NK in infections by *F. tularensis.* Increased IFN-γ response by NK and γδ T cells in infections by *B. pseudomallei*.	[89]
***Renoprotective activity***				
In vivo method	EO fruit extract	Doses of 500 and 1000 mg/kg/day were administered to male adult Wistar albino rats for 15 days.	Reduction of BUN, serum creatinine and renal tissue content of KIM-1. Reduction of levels of MDA, MPO, IFN-γ, caspase-3, collagen IV, endothelin-1 and IL-1.	[90]
In vivo method	Hydroalcoholic EO seed extract	A dose of 200 mg/kg/day was administered to male rats with streptozotocin (STZ)-induced diabetes for 45 days.	Decreased serum levels of urea, creatinine and albumin, renal fibrosis, TBARS, carbonyl and 8-isoprostane levels. No variation in the concentrations of IL-6, TNF-α, MCP-1 and caspase-3. Increase in the number of glomeruli and SOD, catalase and GPx.	[91]
In vivo method	Hydroalcoholic EO seed extract	Young male Winstar rats received 200 mg/kg of the extract for 40 days.	Prevention of the increase in systolic blood pressure, decrease in renal volume, glomeruli and collagen deposition. Decreased serum levels of urea, creatinine and urinary protein. Reduced MDA and carbonyl protein contents, increased nitrite, SOD, CAT and GPx contents.	[92]
***Hematopoietic effect***				
In vivo method	EO fruit pulp	C57BL/6NCrSlc mice were used for dosing and histopathology. A dose of 10 mL/kg/day was tested for 4 days	Increase in erythrocytes, hemoglobin and hematocrit. Increased erythropoietin hormone and transient gene expression hematological factor (EPO) and VEGFA.	[93]

H_2_O_2_: Hydrogen peroxide; ROS: reactive oxygen species; VEGF: vascular endothelial growth factor; VEGFR-2: vascular endothelial growth factor receptor 2; COX-2: cyclooxygenase 2; PGE2: prostaglandin E2; IL-10: interleukin-10; MPO: myeloperoxidase; TNF-α: tumor necrosis factor α; IL-1β: interleukin-1β; IL-6: interleukin-6; PCNA: proliferating cell nuclear antigen; Bcl-2: B-cell lymphoma 2; BAD: anti-Bcl-2-associated; Dlc1: Dlc1Rho GTPase activating protein; Akt3: Akt serine/threonine kinase 3; DNA: deoxyribonucleic acid. DPPH: 2,2-diphenyl-1-picrylhydrazyl; ABTS: 2,2’-azinobis-3-ethylbenzothiazoline-6-sulfonic acid; FRAP: Ferric Reducing Ability; ORAC: Oxygen Radical Absorbance Capacity; IL-1α: interleukin-1α; IL-3: interleukin-3; IL-4: interleukin-4; IL-13: interleukin-13; GABA: Gamma-aminobutyric acid; HBSS: Hank’s balanced salt solution; TERT mRNA: telomerase reverse transcriptase. GLP-1: glucagon-like peptide; eNOS: nitric oxide synthase; SOD1: superoxide dismutase 1; SEAP: secreted embryonic alkaline phosphatase; LPS: lipopolysaccharide; ox-LDL: Low Density Lipoproteins. NLRP3: nod-like receptor pyrin containing 3; p38-MAPK: p38 mitogen-activated protein kinase; NF-κB: nuclear factor κB; iNOS: nitric oxide synthase. N/D: no data; ABCGs: ATP-binding cassette, subfamily G transporters; LDL-R: low-density lipoprotein receptor. L-ORACFL: lipophilic oxygen radical absorbance capacity; COX-1: cyclooxygenase-1. MMP-1: matrix metalloproteinase 1. SREBP-1c: sterol-regulatory-element binding protein-1c; HMG-CoA reductase: 3-hydroxy-3-methylglutaryl CoA reductase; pAMPK: phosphorylated adenosine monophosphate-activated protein kinase; pACC: phosphorylated acetyl-CoA carboxylase; ACC: acetyl-CoA carboxylase; ABCG5/ABCG8: ATP-biding cassette, subfamily G transporter 5/8. IFN-γ: interferon-gamma; NK: natural killer cells; BUN: blood urea nitrogen; KIM-1: of kidney injury molecule-1; MDA: malondialdehyde; TBARS: thiobarbituric acid reactive substances; MCP-1: monocyte chemoattractant protein; SOD: superoxide dismutase; GPx: glutathione peroxidase.

**Table 3:** In vitro and in vivo studies reporting the effect of *Euterpe oleracea* Mart. oil.

Model	Plant Part	Assay/Dose	Results	Ref.
***Anticancer***				
In vitro and in vivo methods	EO oil-containing nanoemulsion (NanoA)	14-Weeks-old C57BL/6 female mice were treated with 100 μL of NanoA directly injected into the tumor mass. The experiment was conducted for 15 days.	Application of photodynamic therapy showed death of B16F10 melanoma cells in vitro and of tumor-bearing C57BL/6 mice.	[95]
***Anti-inflammatory activity***				
In vivo method	EO oil from commercialized pulp	Doses of 500, 1000 and 1500 mg/kg of oil were administered to male Swiss rats for 6 days.	Inhibition in the formation of subcutaneous granulomatous tissue, reduction in ear edema, vascular permeability and migration of neutrophils in peritonitis.	[19]
***Antilipemic activity***				
In vivo method	EO oil obtained from commercialized pulp	Winstar rats were treated by gavage for 10 days at an effective dose of 1226 mg/kg.	The oil reduced levels of total cholesterol, triglycerides and direct low-density lipoprotein cholesterol (LDL-c) but increased that of HDL.	[40]
***Antimicrobial activity***				
In vitro method	EO oil from pulp and seeds	Discs impregnated with 10 μL of EO oil from the pulp and seeds were added to media containing *Pseudomonas aeruginosa* and *Staphylococcus aureus.*	Discs containing EO oil showed inhibition halo at first reading (after 24 h) on *S. aureus.* The oil showed no inhibitory effect on *P. aeruginosa*	[23]
In vitro method	EO oil and inclusion complexes with cyclodextrins.	The drug’s modulatory activity was tested at an initial concentration of 1024 μg/mL.	EO oil showed activity against *Enterococcus faecalis*, *S. aureus*, *P. aeruginosa* and *Escherichia coli*. Inclusion complexes with cyclodextrins showed a reduction in the Minimum Inhibitory Concentration of the oil, increasing its activity.	[25]
***Antinociceptive activity***				
In vivo method	EO oil from commercialized pulp.	Doses of 500, 1000 and 1500 mg/kg of oil were administered to male Swiss rats for 6 days.	Reduction of up to 55.58% in the total number of abdominal contortions.	[19]
***Cytotoxic activity***				
In vivo method	EO oil from pulp	EO oil was diluted in vehicle (1% Tween 80) and administered to male Winstar rats by gavage for 14 days at doses of 30, 100 and 300 mg/kg.	Changes in the thyroid gland directly related to the thyroid follicles. Hypertrophy associated with disorganization and alteration in the chemical composition of the colloid. Disorganization of hepatic tissue, alteration in the amount of lipids and vacuoles in the cytoplasm. The oil led to damage in cells and tissues of both organs.	[96]
***Genotoxic activity***				
In vivo method	EO oil from pulp	Oil was diluted in vehicle (1% Tween 80), and male Wistar rats were treated with EO by gavage at doses of 30, 100 and 300 mg/kg, for 14 days.	Peripheral blood leukocytes, liver, bone marrow and testicular cells indicated that the oil had no significant genotoxic effect. No chromosome breakage, aneugenicity, polychromatic erythrocytes were observed, which indicated no perturbation in hematopoiesis.	[97]

**Table 4:** Patents related to pharmaceutical formulations containing *Euterpe oleracea* Mart. for the treatment and prevention of general diseases.

Reference	IPC	Applicant	Inventor /Year/ Country	Compound/Formulation	Indication/ Pharmacological Profile	Route of Administration/Dose	Assay
PI 0418614-1 A2	A61K A61P	Federal University of Rio de Janeiro	Soares de Moura, 2008, Brazil.	Hydroalcoholic extracts of fruits and lumps (gelatin capsules, tablets)	Analgesic action for treatment and prevention of pain in humans and animals	Oral/10–1000 mg	Preclinical
PI 0604281-3 A2	A61K A61P	Soares de Moura	Soares de Moura, 2008, Brazil.	Hydroalcoholic extracts of fruits and lumps (gelatin capsules, tablets)	Vasodilatory action in the treatment and prevention of vasospastic, ischemic diseases and hypertension.	Oral/10–1000 mg	Preclinical
WO 2011/1036448 A1	A61K A61P	State University of Rio de Janeiro	Soares de Moura, 2011, Brazil.	Hydroalcoholic extracts of the fruits (hydrophobic and hydrophilic ointments)	Accelerated wound healing process.	Topical/0,001 - 100 mg/g.	N/D
US 61/814791	A61K A61P	Neocutis S. A.	Dreher, 2013, USA.	Combined vitamin C, E and an antioxidant compounds, especially EO berries (hydrophobic and hydrophilic formulations for topical use)	Treatment and prevention of skin damage.	Topical and subcutaneous.	N/D
BR 102015017543-4 A2	A61K A61P	Federal University of Maranhão	Nascimento, 2018, Brazil.	Hydroalcoholic extracts obtained from lumps (gelatin capsules, syrups, energy bars and flour)	Chemotherapeutic and chemopreventive activity.	Oral/10, 20 or 40 µg/ml.	Preclinical
BR 102017007451-0 A2	A61K A61P	Federal University of Amapá	Ribeiro da Silva et al. 2018, Brazil.	Ethanolic extract of the fruits (tablet, capsule, granules, controlled release pharmaceutical form).	Antioxidant activity	Oral.	Preclinical
BR 102018005450 3 A2	A61K A61P	Moreira Castilho et al.	Moreira Castilho et al., 2018, Brazil.	Seeds extract (solids, liquids, semisolids or pastes, tablets, hard or soft capsules, lozenges, powders, granules, suspensions, dispersions, emulsions, micro or nanoparticles, liposomes, micelles or vesicles).	Treatment of diseases and metabolic syndromes.	Oral, peroral, enteral, parenteral, topical, transdermal, inhaled, intrapulmonary, vaginal, rectal, intraocular and sublingual.	In vitro
BR 1020180767933	A61K	Federal University of Rio Grande Grande do Norte	Almeida et al. 2018, Brazil.	Inclusion complexes of EO oil and cyclodextrins (tablet, capsule, powder, oral suspension, capsule, cream, ointment or gel).	Infectious processes/ antimicrobial antioxidant and anti-inflammatory activities.	Oral, intravenous, intramuscular, intraperitoneal, subcutaneous, topical.	N/D
BR 102017013494-6 A2	A61K	Federal University of Maranhão	Figueirêdo 2019, Brazil.	Hydroalcoholic extract of the leaves (capsule, solution, syrup, tablets, gel, aerosols, mouthwash, cream, powder, paste, ointment, pellets, suppository and soap)	Infectious processes/ antimicrobial activity (against various bacteria and fungi of clinical interest).	Oral, topical, external and internal use/0,01 to 5g for 100g of product.	N/D

N/D: no data.
